# The opposite prognostic effect of NDUFS1 and NDUFS8 in lung cancer reflects the oncojanus role of mitochondrial complex I

**DOI:** 10.1038/srep31357

**Published:** 2016-08-12

**Authors:** Chia-Yi Su, Yu-Chan Chang, Chih-Jen Yang, Ming-Shyan Huang, Michael Hsiao

**Affiliations:** 1 Genomics Research Center, Academia Sinica, Taipei, Taiwan; 2 Department of Internal Medicine, Kaohsiung Medical University Hospital, Kaohsiung Medical University, Kaohsiung, Taiwan

## Abstract

A recent surge of research on complex I mitochondrial DNA indicates that complex I disassembly regulated by mutation threshold plays a critical role in tumor progression. However, nuclear DNA (nDNA)-encoded core subunits are still a neglected area for cancer investigation. In this study, respective prognostic contributions of 7 nDNA-encoded core subunits were analyzed by immunohistochemical staining and RNA expression data extracted from public resources. The results showed that NDUFS1 and NDUFS8 had the most significant prognostic power in NSCLC patients among all 7 nDNA-encoded core subunits. Patients with low NDUFS1 or high NDUFS8 IHC and RNA expression levels had poor overall survival. Because of the significant correlation between expressions of 7 nDNA-encoded core subunits, multivariate analysis was performed and identified NDUFS1 and NDUFS8 IHC and RNA expression levels retained their leading prognostic roles. By combining NDFUS1 and NDUFS8 as a panel, the most unfavorable prognostic group had a 14-fold increased risk of poor prognosis than the most favorable prognostic group. In conclusion, the opposite prognostic effect of nDNA-encoded core subunits suggests the oncojanus role of nuclear genes regulating complex I dysfunction. The panel with NDUFS1 and NDUFS8 reflecting tumor metabolism status is a novel prognostic predictor for lung cancer.

A cancer cell with its rapid proliferative and aggressive behavior is regarded as a highly metabolic and energy demand biosystem compared to normal cell^1^. Since Warburg effect which stated that cancer cells produce energy predominantly by aerobic glycolysis instead of oxidative phosphorylation proposed over several decades ago, tumor metabolism became a rising field in cancer research^2^,^3^. Mitochondria as the energy factory of the cell is the main place of various energy producing processes and plays an important role in tumor metabolism^4^. From tricarboxylic acid cycle to oxidative phosphorylation, dysfunction of steps in mitochondrial biogenesis cascade alters the balance of intracellular environment during cancer tumorigenesis and progression^5^.

Mitochondrial complex I (NADH dehydrogenase), the first entrance step of oxidative phosphorylation, generates mitochondrial membrane potential for ATP production by converting NADH to NAD + and produces reactive oxygen species (ROS) as byproducts^6^. Complex I as the largest complex in mitochondrial respiratory chain composes of 45 subunits. Among them, 14 subunits form core subunits of complex I, which consists of 7 mitochondrial DNA (mtDNA)-encoded core subunits and 7 nuclear DNA (nDNA)-encoded core subunits^7^. Complex I deficiency caused by complex I gene mutation is known to be the culprit of mitochondrial disorder which leads to neuromuscular symptoms and various clinical presentations^8^. Since cancer is also considered as a mitochondrial disease in a broad definition, there has been a tremendous wave of interest in the relationship between complex I dysfunction and cancer in recent years^9^. Alteration of NAD+/NADH caused by dysfunction of complex I, in turn, activates Akt pathway and inhibits autophagy and results in cancer progression^10^,^11^. Restoring complex I activity or normalizing the NAD+/NADH balance was shown to inhibit cancer metastasis and tumor progression^11^. However, studies focusing on mitochondrial DNA (mtDNA) mutation demonstrated conflicting results and further expanded the debate on the functional role of complex I in cancer. mtDNA mutations are commonly seen in various kinds of cancer due to its vulnerable to the oxidative damage and carcinogens^12^,^13^. Accumulated evidence revealed a paradoxical role of complex I mtDNA mutation in cancer progression^9^. A mutation threshold theory was proposed to determine the pro-tumorigenic or anti-tumorigenic role of mtDNA^14^. Severe mtDNA mutation which led to complex I disassembly induced cell death played a tumor suppression role. In contrast, non-disassembling mtDNA mutation caused tumor growth and metastasis. In addition, heteroplasmic, but not homoplasmic mtDNA mutation, was shown to promote tumor growth^15^. Because of this phenomenon, mitochondrial DNA mutation was named to have an oncojanus function which indicates its double-edged role in tumor progression^14^.

Contrary to the increased attention given to mtDNA mutation in carcinogenesis, the role of nDNA-encoded subunits remains a research field has not yet been much explored. There are 7 nDNA-encoded core subunits including NDUFS1, NDUFS2, NDUFS3, NDUFS7, NDUFS8, NDUFV1, and NDUFV2. Among 7 nuclear DNA-encoded subunits, NDUFS1, NDUFV1, and NDUFV2 belong to NADH dehydrogenase module and NDUFS2, NDUFS3, NDUFS7, and NDUFS8 belong to hydrogenase module^6^. In the present study, the aim was to investigate whether and how complex I nDNA-encoded core subunits have prognostic impact on non-small cell lung cancer patients. The results revealed that low NDUFS1 expression and high NDUFS8 expression are correlated with poor prognosis and have leading prognostic roles in non-small cell lung cancer. The opposite prognostic effect of NDUFS1 and NDUFS8 reflects the oncojanus role of nDNA-encoded core subunits and the panel combining NDUFS1 and NDUFS8 expressions predicts lung cancer prognosis.

## Results

### NDUFS1 and NDUFS8 have the leading prognostic relevance among 7 nDNA-encoded core subunits in NSCLC patients

**Figure 1:**
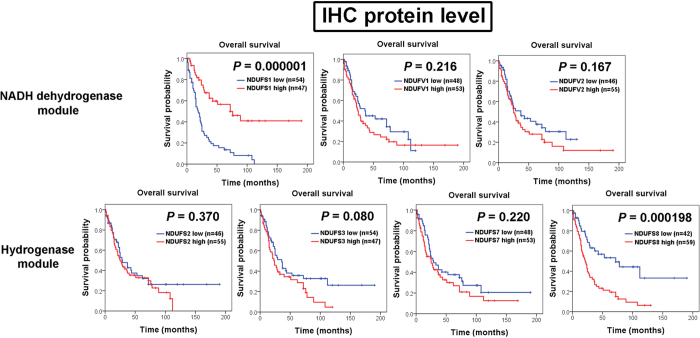
Kaplan-Meier plots of overall survival for IHC expression levels of 7 nuclear DNA-encoded core subunits in 101 non-small cell lung cancer patients. Among NDUFS1, NDUFV1, and NDUFV2 in NADH dehydrogenase module and NDUFS2, NDUFS3, NDUFS7, and NDUFS8 in hydrogenase module, only NDUFS1 of NADH dehydrogenase module and NDUFS8 of hydrogenase module had prognostic significance. Low NDUFS1 expression level (*P* = 0.000001) and high NDUFS8 IHC expression level (*P* = 0.000198) are significantly associated with poorer overall survival.

**Figure 2:**
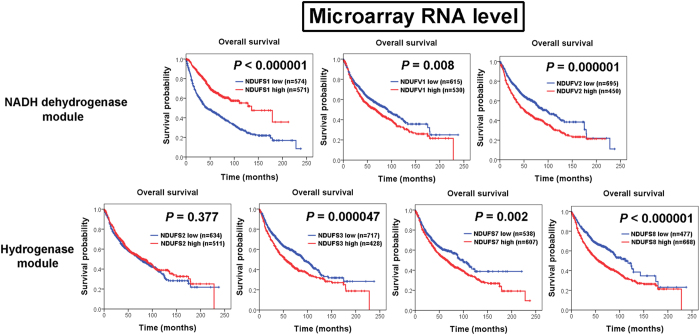
Kaplan-Meier plots of overall survival for RNA expression levels of 7 nuclear DNA-encoded core subunits in 1145 non-small cell lung cancer patients. Among NDUFS1, NDUFV1, and NDUFV2 in NADH dehydrogenase module and NDUFS2, NDUFS3, NDUFS7, and NDUFS8 in hydrogenase module, low NDUFS1 RNA expression level and high NDUFS8 RNA expression level are most significantly associated with poorer overall survival (*P* < 0.000001). High NDUFV1 RNA expression level (*P* = 0.008), high NDUFV2 expression level (*P* = 0.000001), high NDUFS3 expression level (*P* = 0.000047), and high NDUFS7 expression level (*P* = 0.002) are also correlated with poor overall survival.

To evaluate the importance of nDNA-encoded core subunits in lung cancer, we first analyzed the prognostic values of all 7 nDNA-encoded core subunits through immunohistochemistry (IHC) analysis. Interestingly, our results showed that there were differences in prognostic predictive power between 7 nDNA-encoded core subunits. Among all 7 nDNA-encoded core subunits, only NDUFS1 and NDUFS8 IHC expression reached statistical significant correlation with prognosis (Fig. 1). Patients with low IHC expression of NDUFS1 (*P* = 0.000001) and high IHC expression of NDUFS8 (*P* = 0.000198) were found significantly associated with poor overall survival. We further validate the results of our IHC analysis by using Kaplan-Meier plotter web resource. Consistent results were also observed in the survival analysis results of public microarray database. Most significant correlations were observed between low NDUFS1 (*P* < 0.000001) and high NDUFS8 (*P* < 0.000001) RNA expression and poor overall survival (Fig. 2). Patients with high NDUFV1 (*P* = 0.008), NDUFV2 (*P* = 0.000001), NDUFS3 (*P* = 0.000047), and NDUFS7 (*P* = 0.002) RNA expression levels also had poor prognosis values (Fig. 2).

### Only NDUFS1 and NDUFS8 remain their independent prognostic significances in multivariate analysis

Considering that NDUFS1, NDUFS2, NDUFS3, NDUFS7, NDUFS8, NDUFV1, and NDUFV2 being core subunits of mitochondrial complex I, we further analyzed the correlation between their IHC expression levels in clinical NSCLC specimens. Through Spearman’s rank correlation analysis (Supplementary Table 2), significant positive correlations (*P* < 0.01) were seen between the immunoexpression of NDUFV1, NDUFV2, NDUFS2, NDUFS3, NDUFS7, and NDUFS8. Weak positive correlations between NDUFS1 and NDUFV2 (*P* = 0.01) and NDUFS7 (*P* < 0.033) were also noted. Because of the significant correlations between 7 nDNA-encoded core subunits, multivariate analysis was performed to find out if NDUFS1 and NDUFS8 retain their prognostic powers after adjustment for their correlations and pathological stage. In multivariate analysis, NDUFS1 IHC expression (hazard ratio [HR] = 0.297; 95% confidence interval [CI] = 0.167–0.531; *P* = 0.000040), NDUFS8 IHC expression (HR = 2.496; 95% CI = 1.218–5.113; *P* = 0.012), and pathological stage (HR = 4.533; 95% CI = 2.567–8.005; *P* < 0.000001) remained independently correlated with overall survival (Table 1). After false discovery rate correction for multiple testing, NDUFS1 (*P* = 0.01) and NDUFS8 (*P* = 0.04) also retained their prognostic significance (Table 1).

We also analyzed the correlation between RNA levels of all 7 nDNA-encoded core subunits by using extracted data from Kaplan-Meier plotter web resource. There were significant positive correlations (*P* < 0.01) between RNA levels of NDUFV1, NDUFV2, NDUFS2, NDUFS3, NDUFS7, and NDUFS8 (Supplementary Table 3). Significant negative correlations (*P* < 0.01) were seen between NDUFS1 and NDUFV1, NDUFV2, NDUFS3, NDUFS7, and NDUFS8. The results of multivariate analysis of all 7 nDNA-encoded core subunits showed that NDUFS1 RNA expression (HR = 0.510; 95% CI = 0.425–0.613; *P* *<* 0.000001) and NDUFS8 RNA expression (HR = 1.309; 95% CI = 1.045–1.640; *P* = 0.019) were independent prognostic markers for overall survival (Table 2). After false discovery rate correction for multiple testing, NDUFS1 (*P* = 0.01) remained prognostic significant and NDUFS8 had a borderline prognostic significance (*P* = 0.06).

### NDUFS1 IHC expression has significant correlation with T stage, distant metastasis, and pathological stage

**Figure 3:**
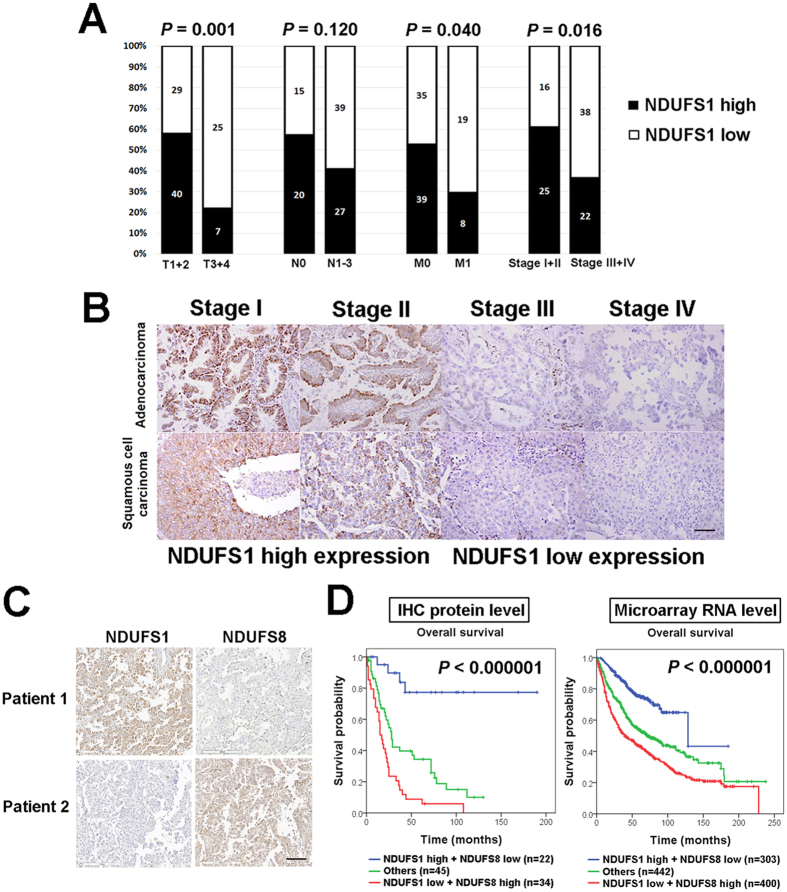
NDUFS1 IHC expression is correlated with pathological stage and the combination of NDUFS1 and NDUFS8 as a panel can predict lung cancer prognosis more precisely. (**A**) In clinicopathological analysis, patients with low NDUFS1 IHC expression have higher T stage, more frequent distant metastasis, and higher pathological stage. (**B**) Representative images of NDUFS1 IHC expression in stage I, stage II, stage III, and stage IV adenocarcinoma and squamous cell carcinoma patients. Photographs were taken at a magnification of 200×. Scale bars represent 100 μm. (**C**) Representative IHC images of two lung cancer patients. Patient 1 has strong NDUFS1 IHC staining and weak NDUFS8 IHC staining and patient 2 with weak NDUFS1 IHC staining and strong NDUFS8 IHC staining. Photographs were taken at a magnification of 200×. Scale bars represent 100 μm. (**D**) The panel comprising NDUFS1 and NDUFS8 stratifies patients into three groups. Patients with low NDUFS1/high NDUFS8 expression have worst prognosis at both IHC and RNA level in Kaplan-Meier survival analysis.

Clinicopathological analysis of NDUFS1 and NDUFS8 indicated that low NDUFS1 IHC expression were significantly correlated with higher T stage (*P* = 0.001), distant metastasis (*P* = 0.040) and higher pathological stage (*P* = 0.016) (Fig. 3A and Supplementary Table 4). However, no significant association between NDUFS8 IHC expression and clinicopathological parameters was seen (Supplementary Table 4). IHC images in Fig. 3B representatively shown loss of NDUFS1 IHC expression in adenocarcinoma and squamous cell carcinoma patients with higher stages.

### Combining NDUFS1 and NDUFS8 IHC expression as a panel reflects the oncojanus role of complex I for prognosis prediction

The opposite prognostic effect of NDUFS1 of NADH dehydrogenase module and NDUFS8 of hydrogenase module revealed in our survival analysis is the clinical evidence of the oncojanus role of complex I. Figure 3C showed representative NDUFS1 and NDUFS8 IHC staining images of two NSCLC cases. Patient 1 had strong NDUFS1 IHC staining and weak NDUFS8 IHC staining and patient 2 with weak NDUFS1 expression and strong NDUFS8 expression. Therefore, we combined NDUFS1 and NDUFS8 IHC expression as a prognostic panel which could further separate patients into 3 groups (Fig. 3D). Patients with low NDUFS1 and high NDUFS8 expression had worst prognosis at both IHC (*P* < 0.000001) and RNA level (*P* < 0.000001). In multivariate analysis, compared to the most favorable prognostic group (high NDUFS1 and low NDUFS8 IHC expression levels), the most unfavorable prognostic group (low NDUFS1 and high NDUFS8 IHC expression levels) showed a 14-fold increased risk of a poor prognosis after adjustment for the pathological stage (Table 3).

## Discussion

Our study evaluated the expression status of 7 nDNA-encoded core subunits in clinical lung cancer patients and the results revealed that different subunits have different or even opposite prognostic effect on lung cancer prognosis. The significant predictive powers of low NDUFS1 expression and high NDUFS8 expression on poor prognosis are the clinical evidence of the oncojanus role of complex I. After adjustment for pathological stage and the correlation between all 7 nDNA-encoded core subunits in multivariate analysis, NDUFS1 and NDUFS8 still are independent prognostic markers for overall survival. With the leading prognostic impact, low NDUFS1 immunoexpression level correlated with higher T stage, distant metastasis, and higher pathological stage in the clinicopathological analysis. Taking together, the panel combining NDUFS1 and NDUFS8 expression could be used to predict the risk of poor prognosis more precisely by reflecting the metabolism status of lung cancer.

Mitochondria has long been considered to have a critical role in cancer progression through regulating the metabolism of rapidly growing tumors^4^. Mitochondrial dysfunctions result from mutations in mitochondrial DNA or nuclear-encoded mitochondrial genes exist in various cancer types^16^. Although not as extensively investigated as mitochondrial DNA mutations, nuclear-encoded mitochondrial gene mutations have gradually been shown to possess cancer-promoting properties^17^. For instance, mutations in succinate dehydrogenase, complex II of oxidative phosphorylation subunits, have association with tumorigenesis of paraganglioma, pheochromocytoma, kidney cancer, gastrointestinal stromal tumor, and breast cancer^18^,^19^,^20^. Besides, mutations in isocitrate dehydrogenase (IDH), an enzyme of tricarboxylic acid cycle, are the most frequent mutations in glioma and can also be found in many kinds of cancer^21^. However, there have been few attempts to clarify the link between complex I nDNA-encoded core subunits and cancer. According to our knowledge, current research is the first study focusing on the role of complex I nDNA-encoded core subunits in lung cancer and unraveled the prognostic power of complex I nDNA-encoded core subunits in cancer patients. Our findings revealed that nDNA-encoded subunits are a critical issue, though it has a relative little discussion in the considerable amount of research in cancer metabolism. A few recent studies on breast cancer shed some light on the relation between complex I nDNA-encoded core subunits and cancer progression. The expression level of NDUFS3 was shown to be positively correlated with nuclear grade in invasive ductal carcinoma and significantly higher in cancer than in normal breast tissue^22^. However, knockdown of NDUFV1 in breast cancer cell lines was demonstrated to increase metastasis activity *in vivo*^11^. This contrary function among the members of nDNA-encoded core subunits also fell into the long debate of oncogenic or tumor suppressive role of mitochondrial dysfunction in cancer^23^,^24^. Different prognostic powers of nDNA-encoded core subunits in our results provided further evidence for the concept of the oncojanus role of complex I dysfunction^25^.

The plausible explanation of this two-sided effect may lie on the structure assembly process of complex I. Research on mtDNA induced complex I disassembly have shown that complex I is essential for cancer cells to enter the process of Warburg effect^26^. Dissembling destruction of complex I could not maintain the basic energy demand for alive in cancer cells and results in an anti-tumorigenic effect. The assembly of complex I composed of 45 subunits is a step by step process in which several subcomplexes enter into the processing by different stage^27^,^28^. Seven nDNA-encoded core subunits could be cluster into 2 functional modules. NADH dehydrogenase module (N module) including NDUFS1, NDUFV1, and NDUFV2 mainly functions on oxidizing NADH and hydrogenase module (Q module) including NDUFS2, NDUFS3, NDUFS7, and NDUFS8 mainly functions on reducing ubiquinone^6^. N module was proposed to enter the assembly process in late stage and Q module enters the process in early stage^29^. Therefore, defects in N module may cause complex I dysfunction without disassembly and lead to cell survival and proliferation. In contrast, defects in Q module may result in complex I disassembly and cell death.

The most powerful independent prognostic effect of NDUFS1 shown in our study reveals the fact that not all nDNA-encoded core subunits have equal prognostic significance. The complicated structure of complex I could also be the explanation for this finding. NDUFS1 as the largest subunit of complex I, its mutations can cause about 80% decrease of complex I activity which implies that NDUFS1 is critical for the stability and function of complex I^30^. Accordingly, loss of NDUFS1 may disrupt the native NADH homeostasis function of complex I and lead to cancer progression. On the other hand, an interesting point worth mention is that, in addition to its native function in tricarboxylic acid cycle, mitochondrial enzyme fumarate hydratase could promote tumor formation through succination of other proteins because of its unique chemical structure^31^,^32^. Although there is no current evidence to suggest that nDNA-encoded core subunits could modulate tumor progression through NADH independent pathway, this possibility cannot be excluded before further investigation.

In conclusion, direct evaluation of the expression levels of nDNA-encoded core subunits in cancer tissue in our study provided the clinical evidence of the oncojanus role of complex I dysfunction, and also revealed that the oncojanus role of complex I dysfunction not only depends on mtDNA but also relies on the regulation of nDNA. NDUFS1 and NDUFS8 as the leading prognostic factors in 7 nDNA-encoded core subunits can be a useful panel for prognosis prediction and therapeutic decision in lung cancer patients.

## Materials and Methods

### Patient and ethics statement

One hundred and one NSCLC formalin-fixed paraffin embedded tissue specimens including 62 cases of adenocarcinoma, 32 cases of squamous cell carcinoma, and 7 cases of large cell carcinoma were retrieved from pathology department archive of Kaohsiung medical university Hospital (Kaohsiung, Taiwan) between 1991 and 2007. All cases were staged according to American Joint Committee on Cancer (AJCC) staging manual and histological cancer types were classified by World Health Organization (WHO) classification. According to the hospital treatment guideline, patients with resectable stage I-III NSCLC received lobectomy or pneumonectomy with mediastinal lymphadenectomy. Patients with resectable stage II and III NSCLC received postoperative adjuvant platinum-based chemotherapy. No adjuvant chemotherapy was administered in patients with completely resected stage I NSCLC. Inoperable locally advanced or metastatic cases were treated with chemotherapy with or without radiotherapy. Overall survival (OS) was defined as the interval from the initial treatment time to death. Clinical information was obtained by retrospectively reviewing medical record. The demographics details of patients are shown in supplementary Table 1. To construct the tissue microarray (TMA), representative 1-mm-diameter core from each case was taken and selected by morphology typical of the diagnosis.

This study was approved by the ethics committees of Institutional Review Board of Kaohsiung Medical University Chung-Ho Memorial Hospital (KMUH-IRB-20110286) and was carried out in accordance with the approved guidelines. No informed consent was required because the data were analyzed anonymously and no identifying information relating to participants were included.

### Immunohistochemical staining and interpretation

IHC staining of 7 nDNA -encoded core subunits including NDUFS1, NDUFV1, NDUFV2, NDUFS2, NDUFS3, NDUFS7, and NDUFS8 were performed on serial sections cut from the TMA. All IHC staining were performed using an automated immunostainer (Ventana Discovery XT autostainer, Ventana, USA). Antigens were retrieved by heat-induced antigen retrieval for 30 minutes with TRIS-EDTA buffer. Slides were stained with polyclonal rabbit NDUFS1 antibody (1:50; Proteintech, USA), polyclonal rabbit NDUFV1 antibody (1:300; GeneTex, Taiwan), polyclonal rabbit NDUFV2 antibody (1:300; GeneTex, Taiwan), polyclonal rabbit NDUFS2 antibody (1:500; GeneTex, Taiwan), polyclonal rabbit NDUFS3 antibody (1:50; Proteintech, USA), polyclonal rabbit NDUFS7 antibody (1:50; GeneTex, Taiwan), and polyclonal rabbit NDUFS8 antibody (1:500; GeneTex, Taiwan),

For IHC staining interpretation, both the immunoreactivity intensity and percentage were recorded. The intensity of staining was defined as 0, no staining; 1+, weak staining; 2+, moderate staining; 3+, strong staining. The extent of staining was scored by the percentage of positive cells (0–100%). The final IHC scores (0–300) were the results of staining intensity score multiplied by the percentage of positive cells. Then, all cases were divided into two groups according to the final IHC scores. A score more than and include 150 itself was defined as high IHC expression level and a score less than 150 was defined as low expression.

### Public database

Public microarray database was used to validate the results from our cohort and to do further analysis. Survival analysis of large cohort microarray database with a total of 1145 NSCLC patients was performed using Kaplan-Meier plotter web resource (http://kmplot.com/analysis/) which includes The Cancer Genome Atlas (TCGA) cohort and multiple GSE datasets from Gene Expression Omnibus (GEO). Raw data including RNA expression levels, survival time, and survival status were extracted from the website for correlation analysis and cox univariate and multivariate analysis.

### Statistical analysis

Statistical analysis was performed with SPSS 20 software (SPSS, Chicago, Illinois, USA). The prognostic impacts of 7 nDNA-encoded core subunits were evaluated by the Kaplan-Meier method and compared by the log-rank test. Follow-up time was censored if the patient was lost during follow-up. Cox univariate and multivariate proportional hazards regression models were also performed with and without the adjustment of the expression levels of 7 nDNA-encoded core subunits and pathological stage. The association between clinicopathological parameters and NDUFS1 and NDUFS8 IHC expression were analyzed by Pearson’s chi-square test. The correlation between the expression levels of 7 nDNA-encoded core subunits was evaluated by Spearman’s rank correlation analysis. For all analyses, a *P* value of <0.05 was considered statistically significant.

## Additional Information

**How to cite this article**: Su, C.-Y. *et al*. The opposite prognostic effect of NDUFS1 and NDUFS8 in lung cancer reflects the oncojanus role of mitochondrial complex I. *Sci. Rep.* **6**, 31357; doi: 10.1038/srep31357 (2016).

## Supplementary Material

Supplementary Information

## Figures and Tables

**Table 1:** Cox univariate and multivariate analysis of overall survival for NDUFS1, NDUFV1, NDUFV2, NDUFS2, NDUFS3, NDUFS7, NDUFS8 IHC expression, and pathological stage in 101 non-small cell lung cancer patients through IHC analysis.

Variables		Cox univariate analysis		Cox multivariate analysis		
		HR(95% CI)	*P*	HR(95% CI)	*P*	FDR-corrected P-value*
NDUFS1 expression	high vs. low	0.294 (0.176–0.492)	0.000003	0.297 (0.167–0.531)	0.000040	0.01
NDUFV1 expression	high vs. low	1.340 (0.836–2.148)	0.223	1.066 (0.607–1.874)	0.823	0.99
NDUFV2 expression	high vs. low	1.391 (0.865–2.236)	0.174	1.171 (0.627–2.187)	0.621	0.99
NDUFS2 expression	high vs. low	1.238 (0.771–1.988)	0.377	0.996 (0.550–1.802)	0.989	0.99
NDUFS3 expression	high vs. low	1.510 (0.944–2.416)	0.085	0.979 (0.513–1.869)	0.949	0.99
NDUFS7 expression	high vs. low	1.335 (0.835–2.134)	0.227	1.418 (0.798–2.521)	0.234	0.47
NDUFS8 expression	high vs. low	2.575 (1.528–4.340)	0.000382	2.496 (1.218–5.113)	0.012	0.04
Pathological stage	III + IV vs. I + II	4.187 (2.474–7.087)	<0.000001	4.533 (2.567–8.005)	<0.000001	0.01

*P value after Benjamini and Hochberg false discovery rate (FDR) procedure.

**Table 2:** Cox univariate and multivariate analysis of overall survival for NDUFS1, NDUFV1, NDUFV2, NDUFS2, NDUFS3, NDUFS7, and NDUFS8 RNA expression in 1145 non-small cell lung cancer patients through public database analysis.

Variables		Cox univariate analysis		Cox multivariate analysis		
		HR(95% CI)	*P*	HR(95% CI)	*P*	FDR-corrected P-value*
NDUFS1 expression	high vs. low	0.457 (0.384–0.545)	<0.000001	0.510 (0.425–0.613)	<0.000001	0.01
NDUFV1 expression	high vs. low	1.248 (1.059–1.470)	0.008	0.955 (0.785–1.163)	0.648	0.75
NDUFV2 expression	high vs. low	1.515 (1.284–1.788)	0.000001	1.155 (0.964–1.382)	0.118	0.16
NDUFS2 expression	high vs. low	0.928 (0.787–1.095)	0.378	0.851 (0.718–1.009)	0.063	0.13
NDUFS3 expression	high vs. low	1.406 (1.192–1.658)	0.000052	1.168 (0.967–1.412)	0.108	0.16
NDUFS7 expression	high vs. low	1.315 (1.109–1.559)	0.002	1.024 (0.845–1.240)	0.812	0.82
NDUFS8 expression	high vs. low	1.636 (1.369–1.955)	<0.000001	1.309 (1.045–1.640)	0.019	0.06
Pathological stage	III + IV vs. I + II	0.457 (0.384–0.545)	<0.000001	0.510 (0.425–0.613)	<0.000001	0.01

*P value after Benjamini and Hochberg false discovery rate (FDR) procedure.

**Table 3:** Cox univariate and multivariate analyses of overall survival in association with NDUFS1 and NDUFS8 IHC expression levels and pathological stage in 101 non-small cell lung cancer patients through IHC analysis.

Variables	Cox univariate analysis		Cox multivariate analysis	
	HR(95% CI)	*P*	HR(95% CI)	*P*
NDUFS1 + NDUFS8				
NDUFS1 high + NDUFS8 low	1		1	
NDUFS1 high + NDUFS8 high	5.365 (1.813–15.877)	0.002	6.753 (2.254–20.235)	0.001
NDUFS1 low + NDUFS8 low	7.403 (2.469–22.198)	0.000353	6.369 (2.109–19.232)	0.001
NDUFS1 low + NDUFS8 high	12.980 (4.532–37.177)	0.000002	14.523 (4.953–42.588)	0.000001
Pathological stage				
I + II	—	—	1	
III + IV	—	—	4.614 (2.629–8.101)	<0.000001
