# Supplementary material for: The opposite prognostic effect of NDUFS1 and NDUFS8 in lung cancer reflects the oncojanus role of mitochondrial complex I
Source: Sci Rep. 2016 Aug 12;6:31357. doi: 10.1038/srep31357 (PMC4981865; doi:10.1038/srep31357)

The opposite prognostic effect of NDUFS1 and NDUFS8 in lung cancer reflects the oncojanus role of mitochondrial complex I

Running title: Prognostic oncojanus roles of NDUFS1 and NDUFS8

Chia-Yi Su[1], Yu-Chan Chang[1], Chih-Jen Yang[2], Ming-Shyan Huang[2*], Michael Hsiao[1*]

[1]Genomics Research Center, Academia Sinica, Taipei, Taiwan

[2]Department of Internal Medicine, Kaohsiung Medical University Hospital, Kaohsiung Medical University, Kaohsiung, Taiwan

*Correspondence to Dr. Michael Hsiao, Genomics Research Center, Academia Sinica, Taipei, Taiwan. Tel: +886-2-2787-1243; Fax: +886-2-2789-9931; E-mail: mhsiao@gate.sinica.edu.tw

or to Dr. Ming-Shyan Huang, Department of Internal Medicine, Kaohsiung Medical University Hospital, Kaohsiung Medical University, Kaohsiung, Taiwan, E-mail: shyang@kmu.edu.tw

Supplementary table 1. Clinicopathological and demographic characteristics of 101 non-small lung cancer patients

Clinicopathological feature	N (%)
All patients	101 (100)
Age	
<65 y	55 (54)
≧65 y	46 (46)
Gender	
Male	57 (56)
Female	44 (44)
Smoking	
Smoker	38 (38)
Non-smoker	63 (62)
Histology subtype	
Adenocarcinoma	62 (61)
Squamous cell carcinoma	32 (32)
Large cell carcinoma	7 (7)
T stage	
T1	20 (20)
T2	49 (48)
T3	6 (6)
T4	26 (26)
N stage	
N0	35 (35)
N1	19 (19)
N2	14 (14)
N3	33 (32)
M stage	
M0	74 (73)
M1	27 (27)
Stage	
I	30 (30)
II	11 (11)
III	31 (30)
IV	29 (29)
Recurrence	
No	25 (25)
Yes	76 (75)

Supplementary Table 2

The correlations between IHC expression levels of NDUFS1, NDUFV1, NDUFV2, NDUFS2, NDUFS3, NDUFS7, and NDUFS8 analyzed by Spearman's rank correlation analysis

	NDUFS1	NDUFV1	NDUFV2	NDUFS2	NDUFS3	NDUFS7	NDUFS8
NDUFS1	1						
NDUFV1	0.053	1					
NDUFV2	0.255*	0.443**	1				
NDUFS2	0.136	0.364**	0.361**	1			
NDUFS3	0.164	0.292**	0.455**	0.494**	1		
NDUFS7	0.212*	0.404**	0.364**	0.404*	0.252*	1	
NDUFS8	-0.099	0.364**	0.439**	0.277**	0.425**	0.404**	1

*. Correlation is significant at the 0.05 level (2-tailed).

**. Correlation is significant at the 0.01 level (2-tailed).

Supplementary Table 3

The correlations between RNA expression levels of NDUFS1, NDUFV1, NDUFV2, NDUFS2, NDUFS3, NDUFS7, and NDUFS8 analyzed by Spearman's rank correlation analysis

	NDUFS1	NDUFV1	NDUFV2	NDUFS2	NDUFS3	NDUFS7	NDUFS8
NDUFS1	1						
NDUFV1	-0.106**	1					
NDUFV2	-0.313**	0.243**	1				
NDUFS2	-0.003	0.220**	0.116**	1			
NDUFS3	-0.153**	0.427**	0.313**	0.171**	1		
NDUFS7	-0.097**	0.355**	0.270**	0.074*	0.395**	1	
NDUFS8	-0.231**	0.518**	0.328**	0.124**	0.470**	0.553**	1

**. Correlation is significant at the 0.01 level (2-tailed).

Supplementary Table 4

Correlations of clinicopathological features of lung cancer patients with NDUFS1 and NDUFS8 IHC expression

Clinicopathological feature	n	NDUFS1 expression, n (%)		*P*	NDUFS8 expression, n (%)		*P*
		Low (n=54)	High (n=47)		Low (N=42)	High (N=59)	
Age							
<65 y	55	24 (43.6)	31 (56.4)	0.030	22 (40.0)	33 (60.0)	0.724
≧65 y	46	30 (65.2)	16 (34.8)		20 (43.5)	26 (56.5)	
Gender							
Male	57	32 (56.1)	25 (43.9)	0.540	26 (45.6)	31 (54.4)	0.350
Female	44	22 (50.0)	22 (50.0)		16 (36.4)	28 (63.6)	
Smoking							
Smoker	38	22 (57.9)	16 (42.1)	0.488	14 (36.8)	24 (63.2)	0.453
Non-smoker	63	32 (50.8)	31 (49.2)		28 (44.4)	35 (55.6)	
Histology							
Adenocarcinoma	62	29 (46.8)	33 (53.2)	0.215	21 (33.9)	41 (66.1)	0.046
Squamous cell carcinoma	32	20 (62.5)	12 (37.5)		19 (59.4)	13 (40.6)	
Large cell carcinoma	7	5 (71.4)	2 (28.6)		2 (28.6)	5 (71.4)	
T stage							
T1+T2	69	29 (42.0)	40 (58.0)	0.001	27 (39.1)	42 (60.9)	0.463
T3+T4	32	25 (78.1)	7 (21.9)		15 (46.9)	17 (53.1)	
N stage							
N0	35	15 (42.9)	20 (57.1)	0.120	18 (51.4)	17 (48.6)	0.144
N1-N3	66	39 (59.1)	27 (40.9)		24 (36.4)	42 (63.6)	
M stage							
M0	74	35 (47.3)	39 (52.7)	0.040	34 (45.9)	40 (54.1)	0.141
M1	27	19 (70.4)	8 (29.6)		8 (29.6)	19 (70.4)	
Pathological stage							
I + II	41	16 (39.0)	25 (61.0)	0.016	19 (46.3)	22 (53.7)	0.423
III + IV	60	38 (63.3)	22 (36.7)		23 (38.3)	37 (61.7)	

Supplementary figure legends

Supplementary figure 1. Representative images showing the intensity of the immunostaining of NDUFS1, NDUFS2, NDUFS3, NDUFS7, NDUFS8, NDUFV1, and NDUFV2 in the non-small lung cancer tissue microarrays. The images were taken at a magnification of 200×. Scale bars represent 100 μm.

Supplementary Figure 1

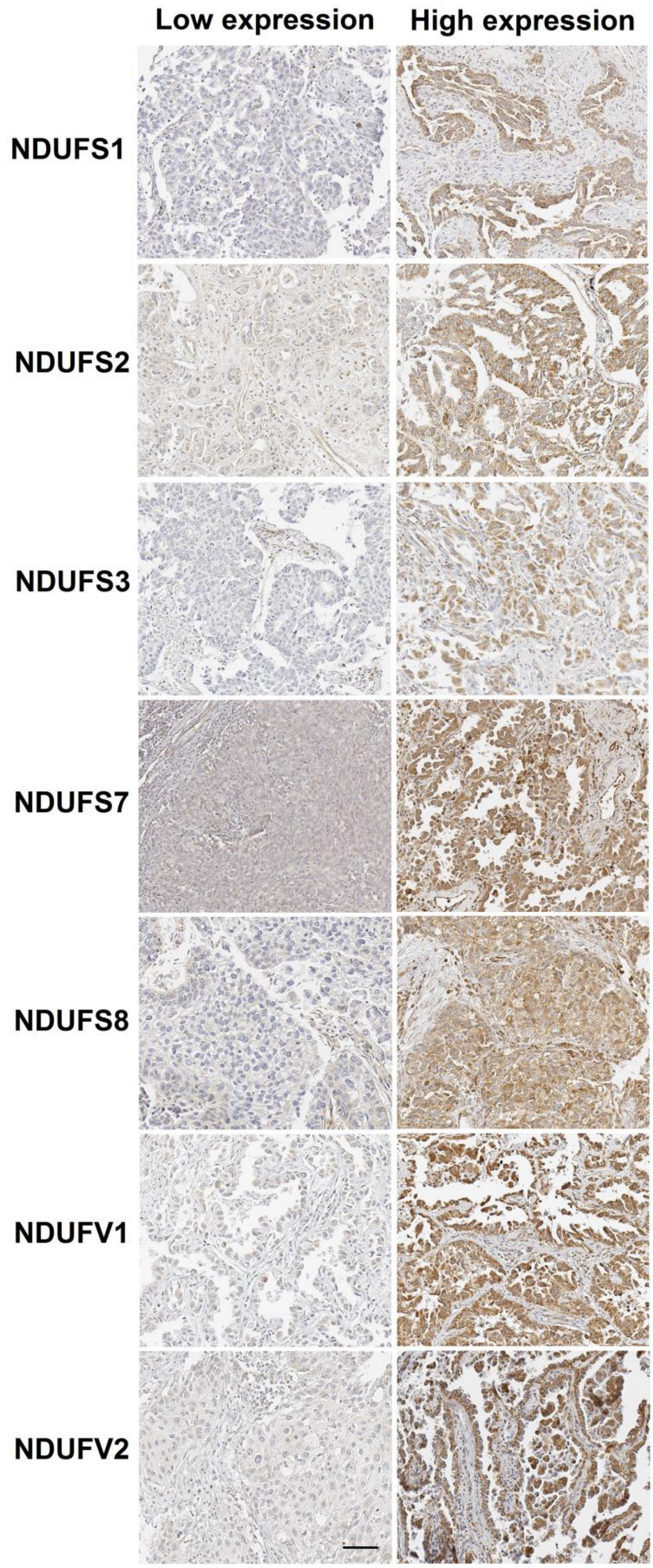

Supplement: Supplementary Information [file srep31357-s1.pdf]
